# Rubidium chloride modulated the fecal microbiota community in mice

**DOI:** 10.1186/s12866-021-02095-4

**Published:** 2021-02-15

**Authors:** Qian Chen, Zhiguo He, Yuting Zhuo, Shuzhen Li, Wenjing Yang, Liang Hu, Hui Zhong

**Affiliations:** 1 School of Life Sciences, Central South University, Changsha, 410013 China; 2 School of Minerals Processing and Bioengineering, Key Laboratory of Biohydrometallurgy of Ministry of Education, Central South University, Changsha, 410083 China

**Keywords:** Fecal microbial community, Rubidium (Rb), Microbiome, Anticancer, Anti-depressant

## Abstract

**Background:**

The microbiota plays an important role in host health. Although rubidium (Rb) has been used to study its effects on depression and cancers, the interaction between microbial commensals and Rb is still unexplored. To gain the knowledge of the relationship between Rb and microbes, 51 mice receiving RbCl-based treatment and 13 untreated mice were evaluated for their characteristics and bacterial microbiome changes.

**Results:**

The 16S ribosomal RNA gene sequencing of fecal microbiota showed that RbCl generally maintained fecal microbial community diversity, while the shifts in fecal microbial composition were apparent after RbCl exposure. RbCl significantly enhanced the abundances of *Rikenellaceae*, *Alistipes*, *Clostridium XlVa* and sulfate-reducing bacteria including *Deltaproteobacteria*, *Desulfovibrionales*, *Desulfovibrionaceae* and *Desulfovibrio*, but significantly inhibited the abundances of *Tenericutes*, *Mollicutes*, *Anaeroplasmatales*, *Anaeroplasmataceae* and *Anaeroplasma* lineages. With regarding to the archaea, we only observed two less richness archaea *Sulfolobus* and *Acidiplasma* at the genus level.

**Conclusions:**

Changes of fecal microbes may in part contribute to the anticancer or anti-depressant effects of RbCl. These findings further validate that the microbiome could be a target for therapeutic intervention.

**Supplementary Information:**

The online version contains supplementary material available at 10.1186/s12866-021-02095-4.

## Background

Rubdium (Rb) is found in air, soil, water and organisms, and is a less studied alkali metal element and can efficiently transfer to the human body through the food chain (soil-plant-human) [1]. Since the first report on its correlation with phenylketonuria and maple-syrup-urine disease [2], some studies have suggested its effects on tumor [3, 4], depression [5–7] and cardiovascular system [8].

Researchers found that many cancers were caused by the changes of Rb^+^ levels in the body [3, 9]. Some other studies have shown that Rb was easily taken up by cancer cells and might affect the proliferation of cancer cells [4, 10]. There are several studies reporting that Rb could be used to treat depression [11, 12]. Later studies confirmed that Rb decreased the depression-like behavior via nitric oxide (NO) pathway [7]. Although there are some hypotheses about the mechanisms of anticancer or anti-depressant of Rb, none of them provided sufficiently reliable evidence.

The microbiome is a dynamic ecological community which mainly includes bacteria, archaea, fungi and viruses [13]. There is growing evidence proving that the microbiome plays key roles in the cancer and neurological disease [14–17]. During recent years, the potential role of the microbiome in various human diseases has attracted the attention of researchers [13, 18]. Many diseases, including cancer and depression, are related to the imbalance of the microbial community. Recent studies reported that the microbial community of colorectal cancer (CRC) patients was significantly different from that of healthy individuals [19]. These specific species of the microbiota, such as *Fusobacterium nucleatum*, *Enterococcus faecalis*, *Bacteroides fragilis*, and *Escherichia coli*, etc., were enriched in the stool of CRC patients and promoted the development of CRC [20]. Some researchers reported that specific microorganisms, including *Firmicutes* and *Bacteroidetes*, might be involved in the occurrence and the development of depression [21]. Meanwhile, *Lactobacillus* and *Bifdobacterium* were reported to be beneficial to the treatment of depression [22, 23].

However, to our best knowledge, no work has been reported on the effect of chemical element Rb on the microbiome, and whether Rb inhibits tumor and depression thorough changing the community composition of microbiome is still not clarified. Therefore, the present study was to investigate the relationship between the addition of rubidium chloride (RbCl) and composition of fecal microbiome in order to further understand the mechanism of Rb against cancer and neurological disease from the perspective of fecal microbial community.

## Results

### Effect of RbCl on animal characteristics

**Fig. 1:**
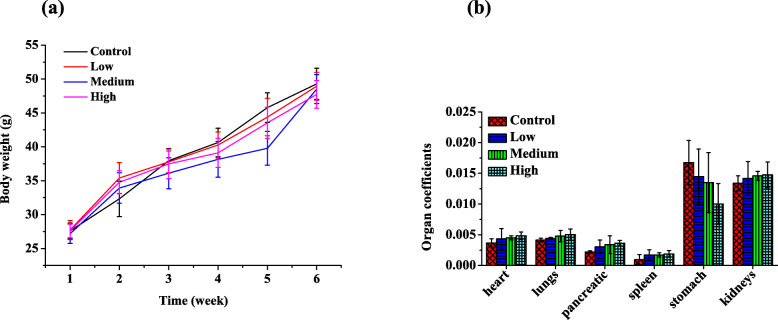
Effects of rubidium chloride on the mice characteristics in control, low-dose (20 mg/L), middle-dose (50 mg/L) and high-dose groups (100 mg/L). **a** Body weight. **b** Organ coefficients. Values are means ± SD; Control, *n* = 13; Low, *n* = 17; Medium, *n* = 17; High, *n* = 17. *P < 0.05, **P < 0.01, ***P < 0.001 when compared with control

To better understand the effects of RbCl on mice, we conducted a follow-up study of 64 mice and recorded body weights and multiple organ weights of each mouse. Changes of the body weights in all the groups were shown in Fig. 1a. The weights of mice in the drug groups decreased when compared with mice in control group and were negatively correlated with dosage whereas these differences were not statistically significant. Additionally, multiple organ coefficients were observed (Fig. 1b). Interestingly, as RbCl concentration was increased, the organ coefficient of stomach gradually decreased. However, there was an increase in organ coefficients among other organs such as pancreatic, spleen, kidneys, lungs and heart. Changes of gastric organ coefficient during drug administration could be explained by route of administration. These data indicated that RbCl had little effect on animal characteristics.

### Effect of RbCl on the fecal microbial communities

**Fig. 2:**
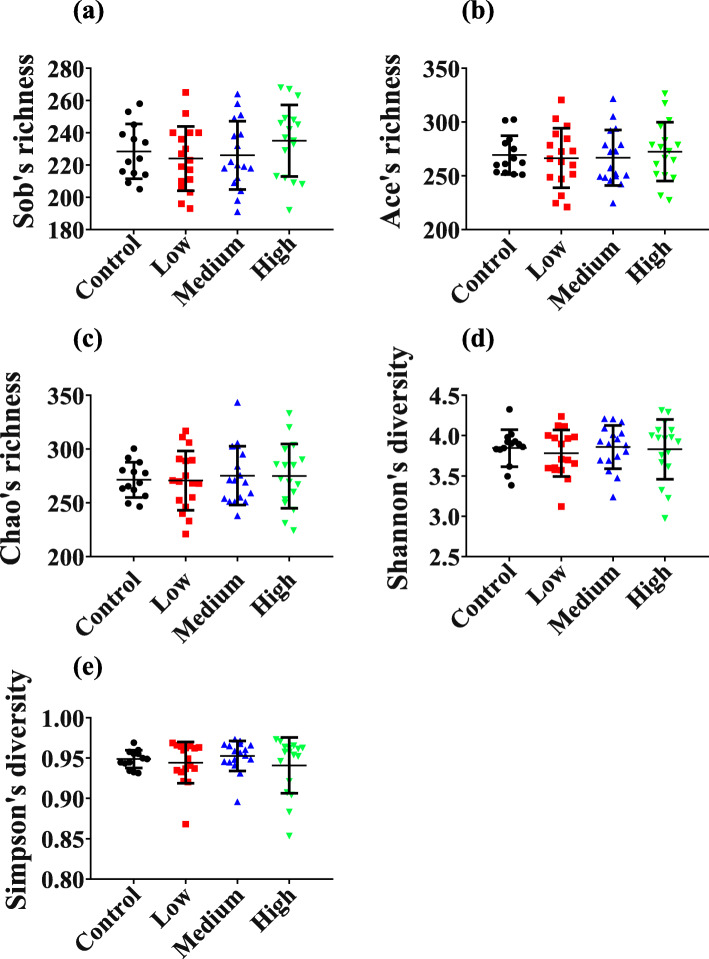
The diversity and richness of samples collected from 64 mice; **a** Sob’s richness; **b** Ace’s richness; **c** Chao’s richness; **d** Shannon’s diversity; **e** Simpson’s diversity

**Fig. 3:**
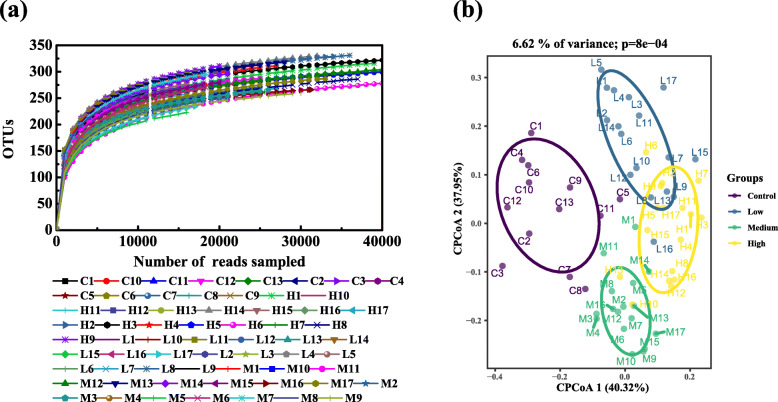
Variations of microbial communities in four groups. **a** Rarefaction curves of the samples; **b** Constrained PCoA plots of Bray-Curtis distances among the four groups

From the results of 16S rRNA gene sequencing, we obtained a total of 1,481,388 high-quality reads for 64 fecal samples of four groups, which could be clustered into 486 OTUs. Figure 3a indicates the rarefaction curves of all samples. The curves tended to be flat as the number of extracted sequences increased, indicating that the sequencing depths included most of the microbes in samples. As shown in Fig. 2, the indices reflecting community richness include Sobs, Chao and Ace. The indices reflecting community diversity are Shannon and Simpson. The richness and diversity indexes demonstrated no statistical differences in control, low-dose (Chao, *P* = 0.9401; Sobs, *P* = 0.5239; Ace, *P* = 0.7497; Shannon, *P* = 0.5082; Simpson, *P* = 0.5401), middle-dose (Chao, *P* = 0.6578; Sobs, *P* = 0.7346; Ace, *P* = 0.7640; Shannon, *P* = 0.8858; Simpson, *P* = 0.5176) and high-dose groups (Chao, *P* = 0.7105; Sobs, *P* = 0.3809; Ace, *P* = 0.7243; Shannon, *P* = 0.8954; Simpson, *P* = 0.4176). In addition, to assess the effect of the different treatments on the assembly of bacterial communities, we compared the β-diversity (between-samples diversity) using Bray Curtis distances and performed constrained principal coordinate analysis (CPCoA). This analysis revealed a clear differentiation of samples belonging to the control, low-dose, middle-dose, and high-dose groups that explained as much as 6.62% of the overall variance of the data (Fig. 3b; *P* < 0.001). Thus, the above results showed that RbCl did not affect the diversity and richness of the fecal microbial community in general. However, it altered the structure of the fecal bacterial community, reflected in changes in fecal microbial composition.

### Effect of RbCl on fecal bacterial composition

**Fig. 4:**
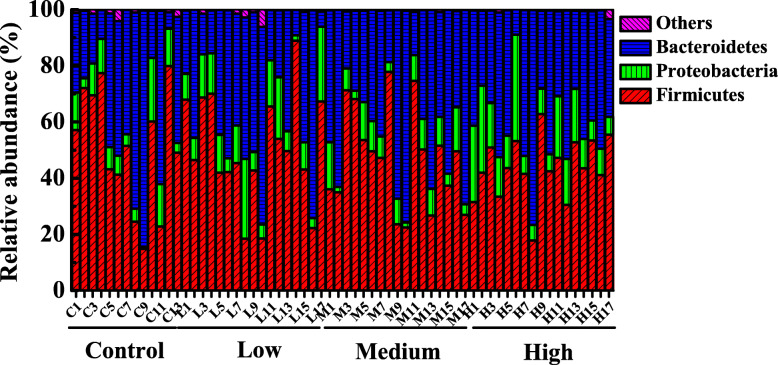
Relative abundance of fecal microorganisms at the phylum level, different colors represent different microbe. “Others” represents the microbes with relative abundance less than 5%

**Fig. 5:**
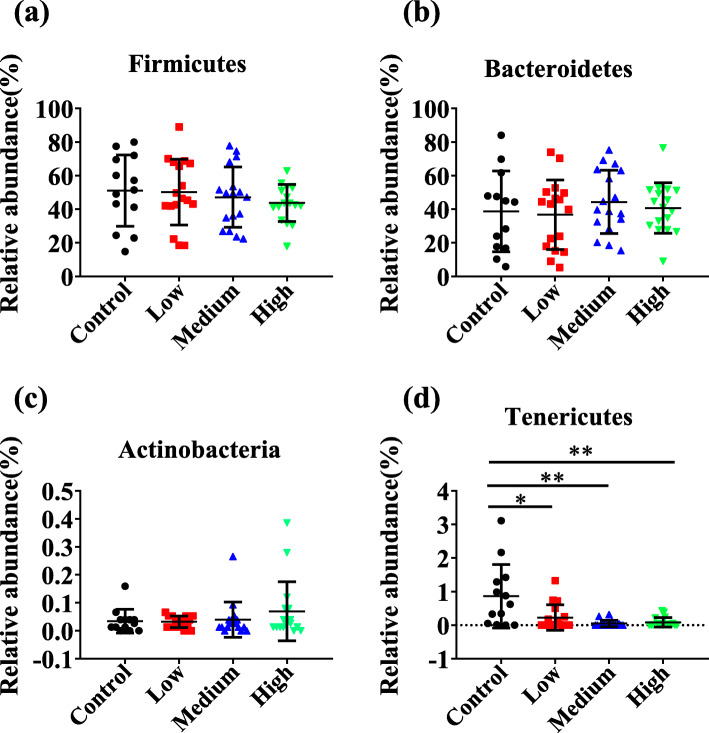
Statistical analysis of relative abundance of fecal bacteria at the phylum level. **a** The abundances of *Firmicutes* were not significantly altered among four groups. **b** The abundances of *Bacteroidetes* showed no statistical differences among four groups. **c** The abundances of *Actinobacteria* showed no statistical differences among four groups. **d** The abundances of *Tenericutes* were significantly lower in RbCl groups (*P* < 0.05) than the control group. Data are shown as mean ± SD. *P < 0.05, **P < 0.01, ***P < 0.001

All OTUs were clustered into 12 phyla, 19 classes, 27 orders, 44 families, 92 genera. The venn diagram (Fig. S1) showed 352 shared OTUs among all the fecal samples, and samples in control, low-dose, middle-dose and high-dose groups had 7, 14, 8 and 10 unique OTUs, respectively. Results indicated that although the proportion of shared microbial communities was very high, distinct microbial communities still existed in different treatment groups. Compositions of fecal bacteria in all samples were determined using 16S rRNA gene sequencing. The fecal microbial compositions of phylum with relative abundance above 5% were seen in Fig. 4. Other microorganisms with relative abundance less than 5% were shown in Table. S1. In all samples, *Firmicutes* was the dominant phylum with average abundances of 51.03, 50.18, 47.15 and 43.73% in control, low-dose, middle-dose and high-dose groups, respectively (Fig. 5a). The relative abundances of *Bacteroidetes*, the second dominant phylum, were not significantly different among the four groups (Fig. 5b). Moreover, less richness *Tenericutes* (the average abundances were 0.86, 0.23, 0.05 and 0.08%) and *Actinobacteria* (the average abundances were 0.03, 0.03, 0.04 and 0.07%) were observed in control, low-dose, middle-dose and high-dose groups, respectively (Table. S1). As shown in Fig. 5c and d, enrichment of *Actinobacteria* and depletion of *Tenericutes* (*P* < 0.01) were correlated with high doses of RbCl.

**Fig. 6:**
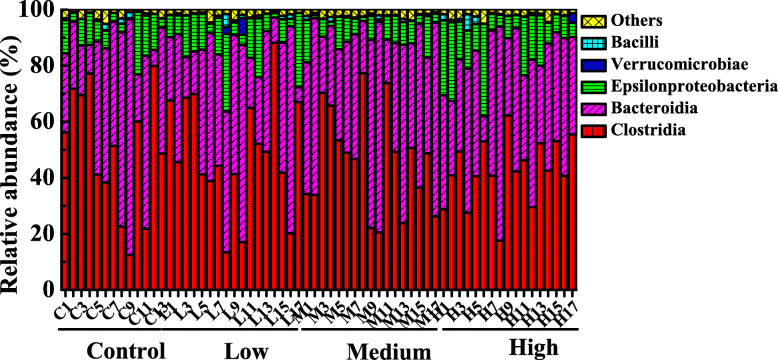
Relative abundance of fecal microorganisms at the class level, different colors represent different microbe. “Others” represents the microbes with relative abundance less than 5%

**Fig. 7:**
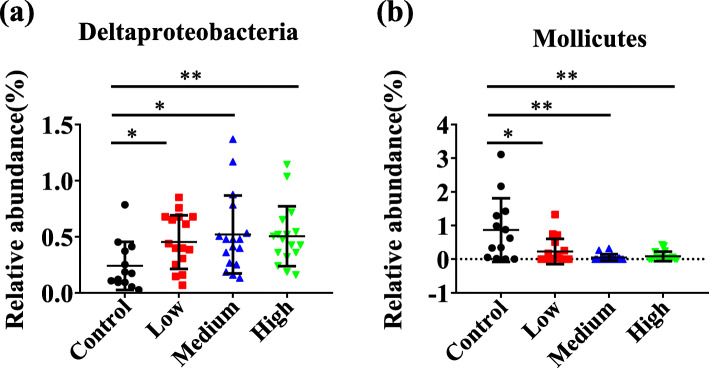
Statistical analysis of relative abundance of fecal bacteria at the class level. **a** The abundances of *Deltaproteobacteria* were significantly increased in RbCl groups compared with the control group (*P* < 0.05). **b** The abundances of *Mollicutes* were significantly decreased in RbCl groups compared with the control group (*P* < 0.05). Data are shown as mean ± SD. *P < 0.05, **P < 0.01, ***P < 0.001

The fecal microorganisms from four groups were separated into 3 dominant classes including *Bacteroidia*, *Clostridia* and *Epsilonproteobacteria* (Fig. 6). Other classes with relative abundance less than 5% were shown in Table S2. Statistically significant differences between the experimental groups and the control group were also performed in our study. The abundances of *Deltaproteobacteria* were significantly higher in three experimental groups (*P* < 0.05) than those of the control (Fig. 7a). In addition, differences in the relative abundances of *Mollicutes* were significant in control, low-dose (*P* = 0.0175), middle-dose (*P* = 0.0014) and high-dose groups (*P* = 0.0022) (Fig. 7b).

**Fig. 8:**
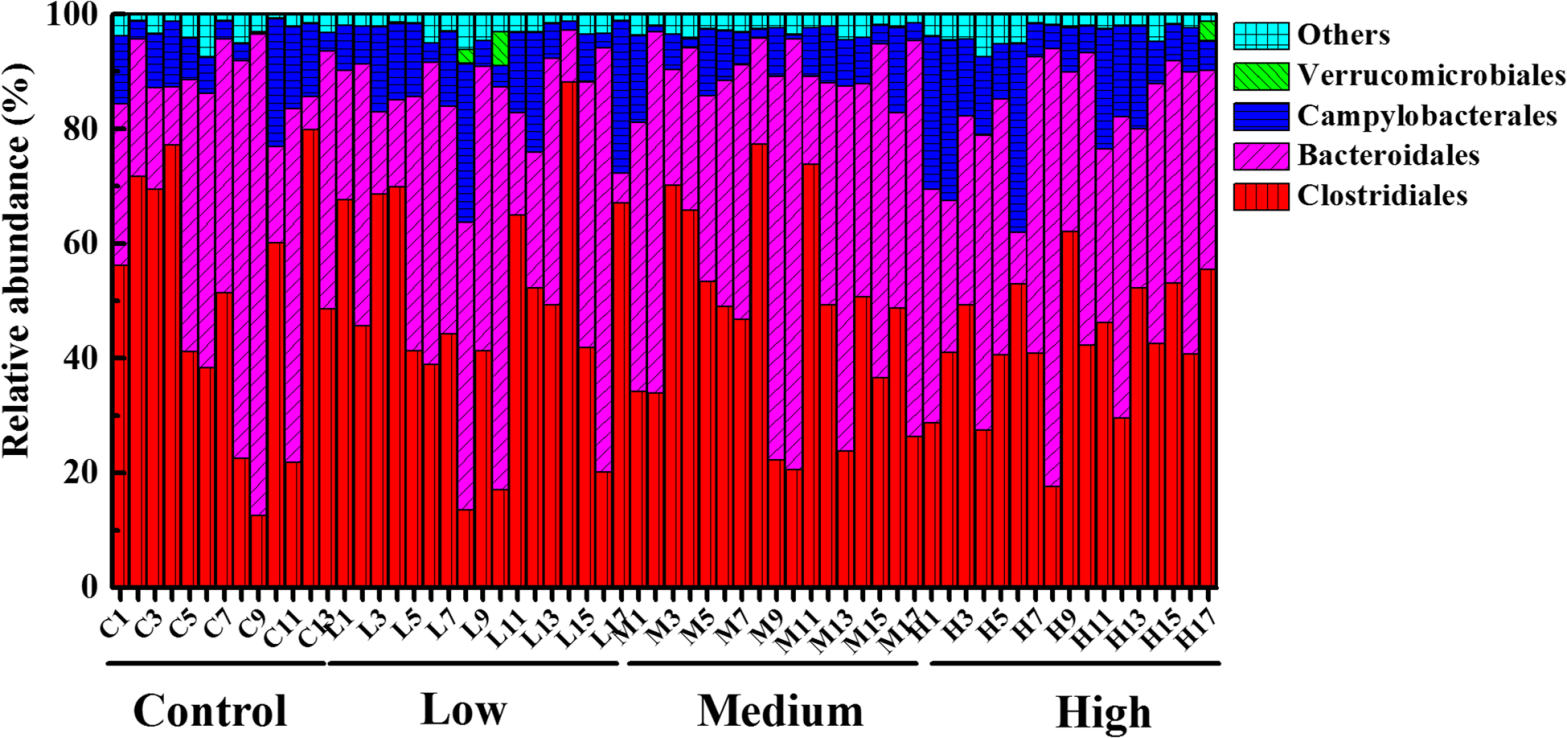
Relative abundance of fecal microorganisms at the order level, different colors represent different microbe. “Others” represents the microbes with relative abundance less than 5%

**Fig. 9:**
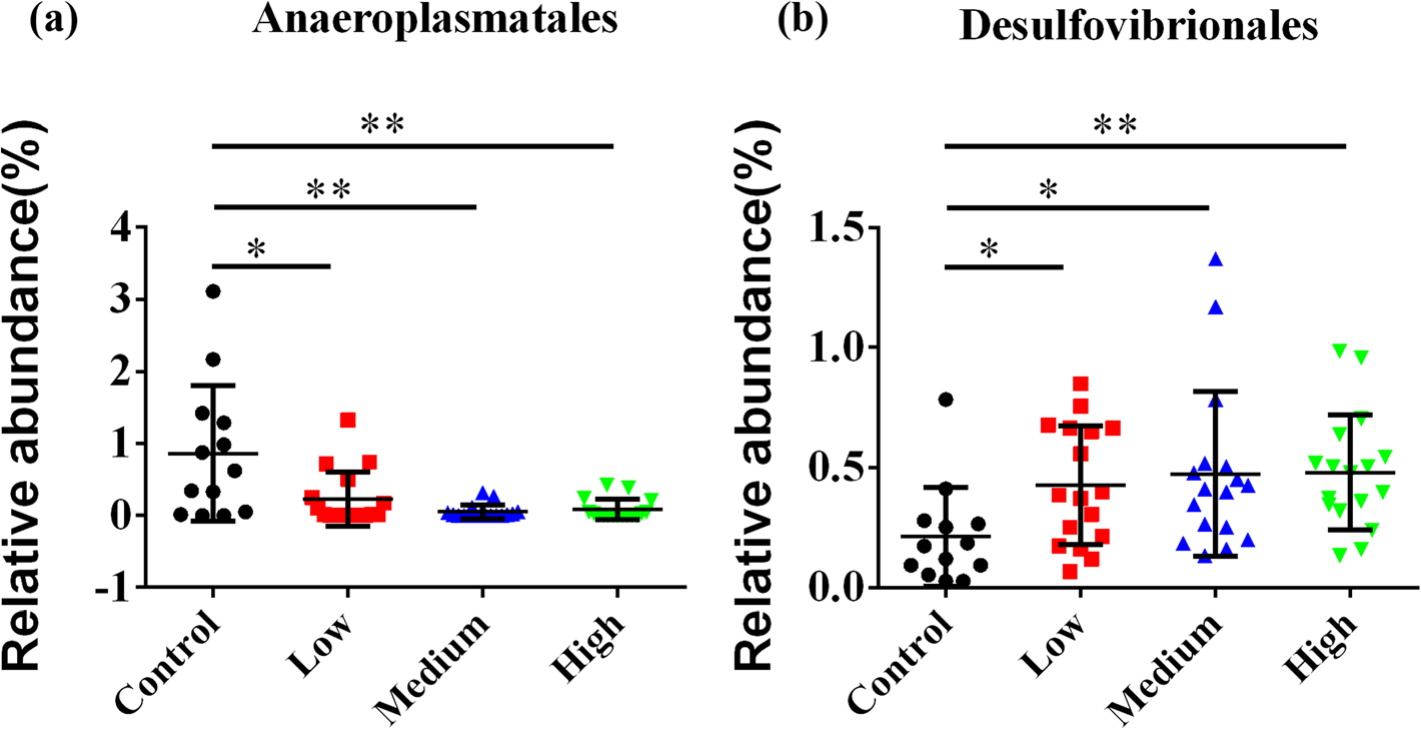
Statistical analysis of relative abundance of fecal bacteria at the order level. **a** The abundances of *Anaeroplasmatales* were significantly decreased in RbCl groups compared with the control group (*P* < 0.05). **b** The abundances of *Desulfovibrionales* were significantly increased in RbCl groups compared with the control group (*P* < 0.05). Data are shown as mean ± SD. *P < 0.05, **P < 0.01, ***P < 0.001

The fecal microbes with relative abundance above 5% at the level of order were shown in Fig. 8**.** A total of 27 orders were observed in all samples (Table. S3**)**. The relative proportion of *Anaeroplasmatales* was significantly increased in control group (*P* < 0.05) (Fig. 9a), while the abundance of *Desulfovibrionales* was significantly higher in low-dose (*P* = 0.0176), middle-dose (*P* = 0.0219) and high-dose groups (*P* = 0.0033) than control group (Fig. 9b).

**Fig. 10:**
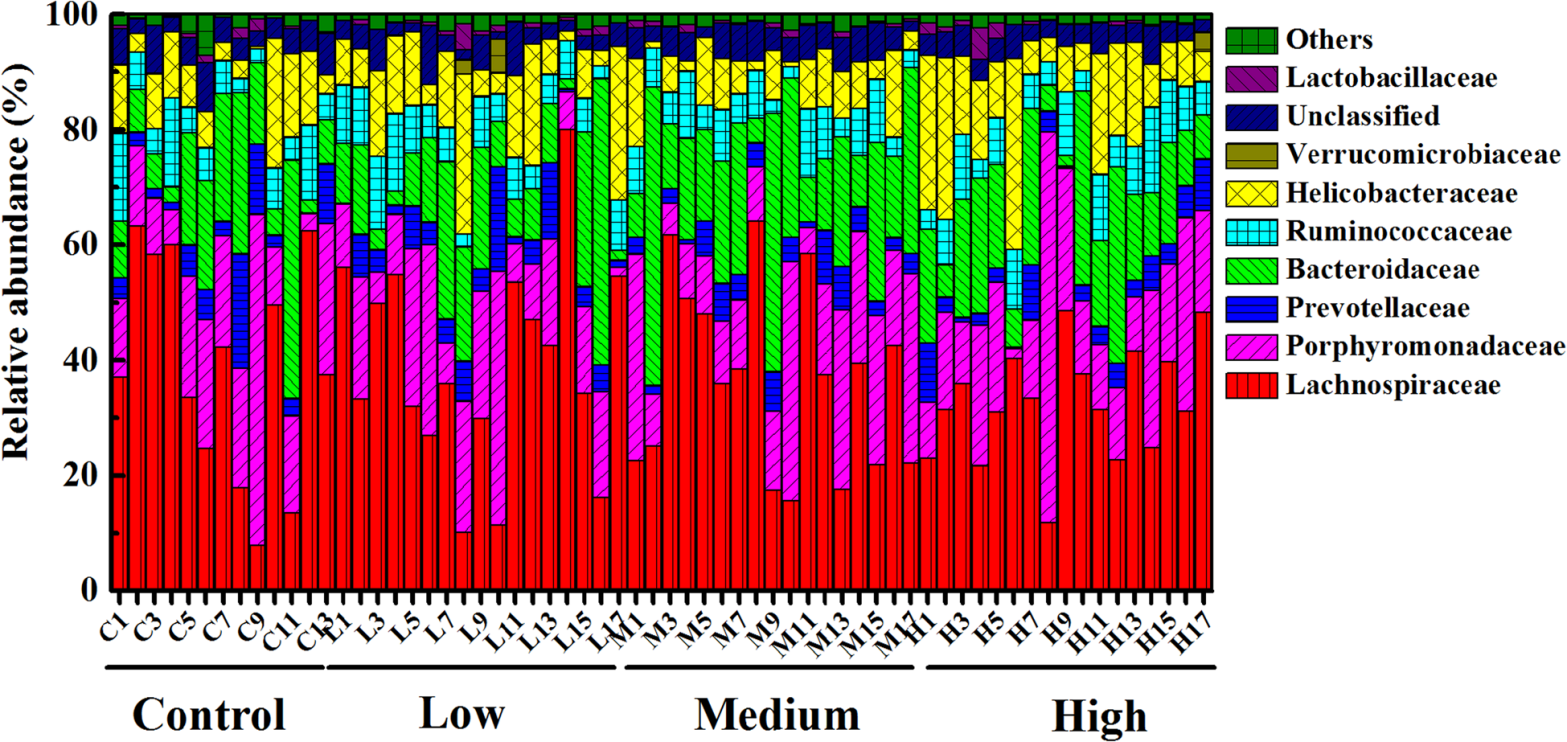
Relative abundance of fecal microorganisms at the family level, different colors represent different microbe. “Others” represents the microbes with relative abundance less than 5%

**Fig. 11:**
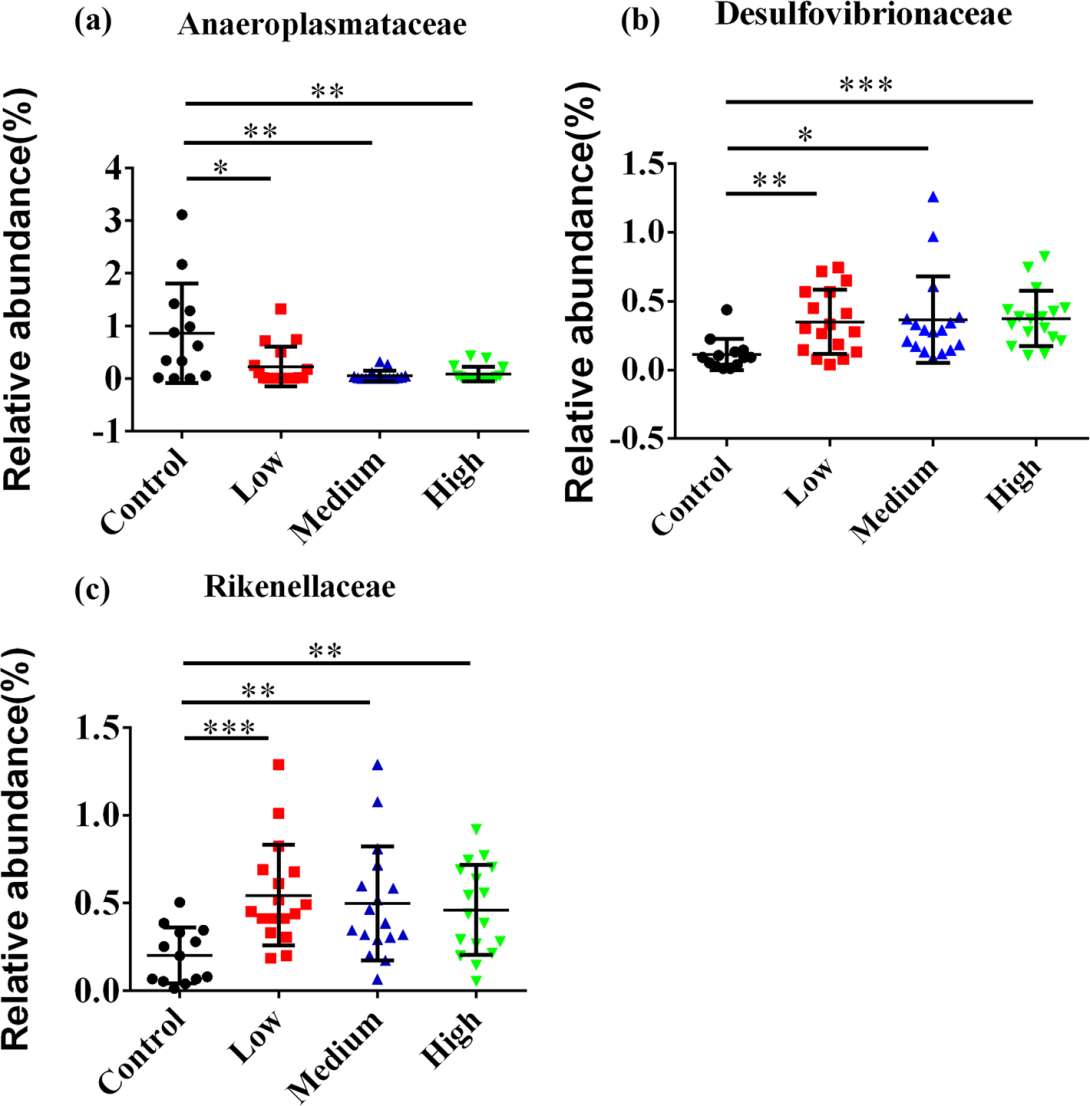
Statistical analysis of relative abundance of fecal bacteria at the family level. **a** The abundances of *Anaeroplasmataceae* were significantly decreased in RbCl groups compared with the control group (*P* < 0.05). **b** The abundances of *Desulfovibrionaceae* were significantly increased in RbCl groups compared with the control group (*P* < 0.05). **c** The abundances of *Rikenellaceae* were significantly increased in RbCl groups compared with the control group (*P* < 0.05). Data are shown as mean ± SD. *P < 0.05, **P < 0.01, ***P < 0.001

At the family level, fecal microbes with relative abundance above 5% were shown in Fig. 10**.** Others families with relative abundance less than 5% were observed in Table. S4. Among these families, the abundance of *Anaeroplasmataceae* was found significantly higher in control group (*P* < 0.05) than three experimental groups (Fig. 11a), while the abundances of *Desulfovibrionaceae* were significantly increased in three experimental groups (*P* < 0.05) (Fig. 11b). Besides, compared with the control group, the abundances of *Rikenellaceae* significantly increased in low-dose (*P* < 0.0006), middle-dose (*P* < 0.0054) and high-dose groups (*P* < 0.0033) (Fig. 11c).

**Fig. 12:**
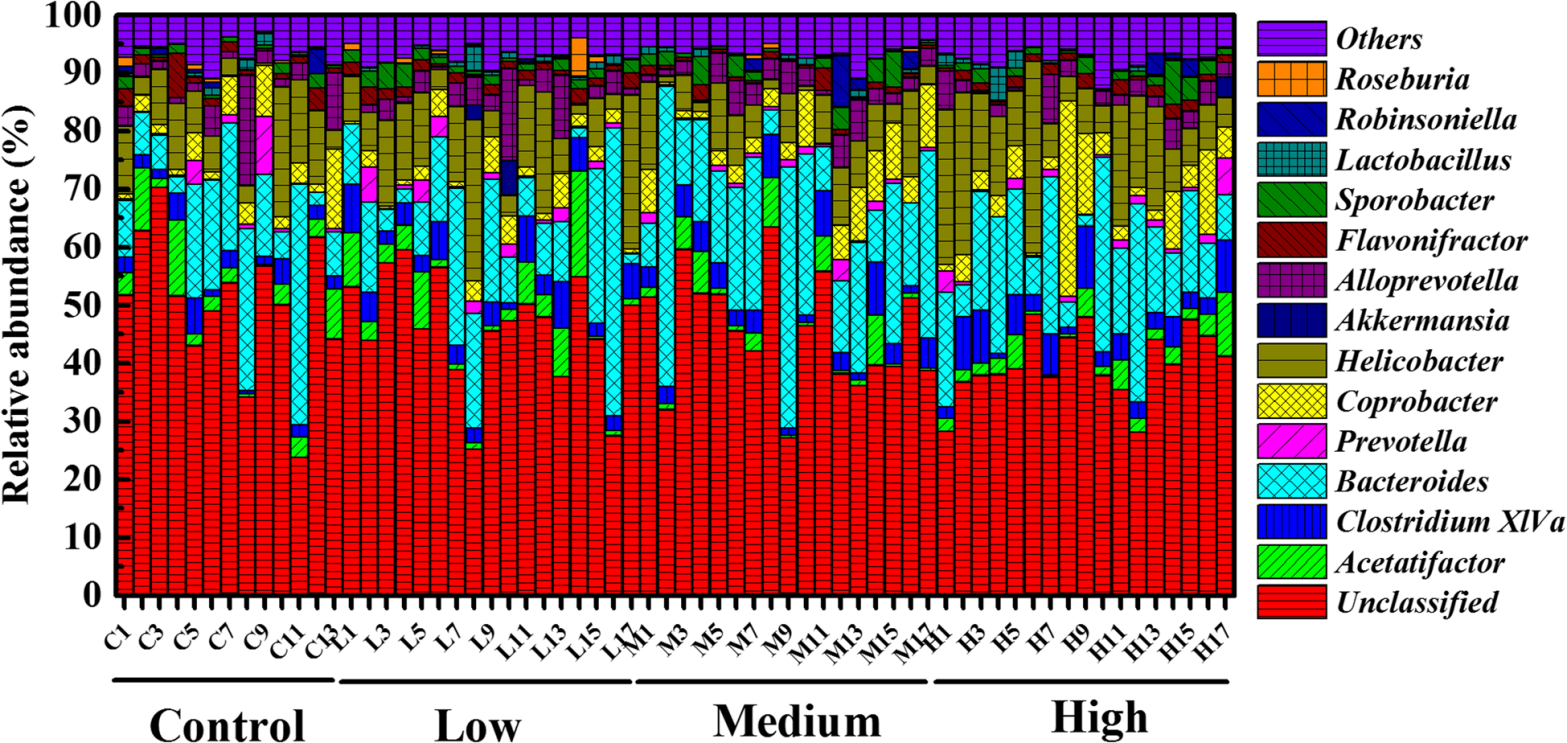
Relative abundance of fecal microorganisms at the genus level, different colors represent different microbe. “Others” represents the microbes with relative abundance less than 5%

**Fig. 13:**
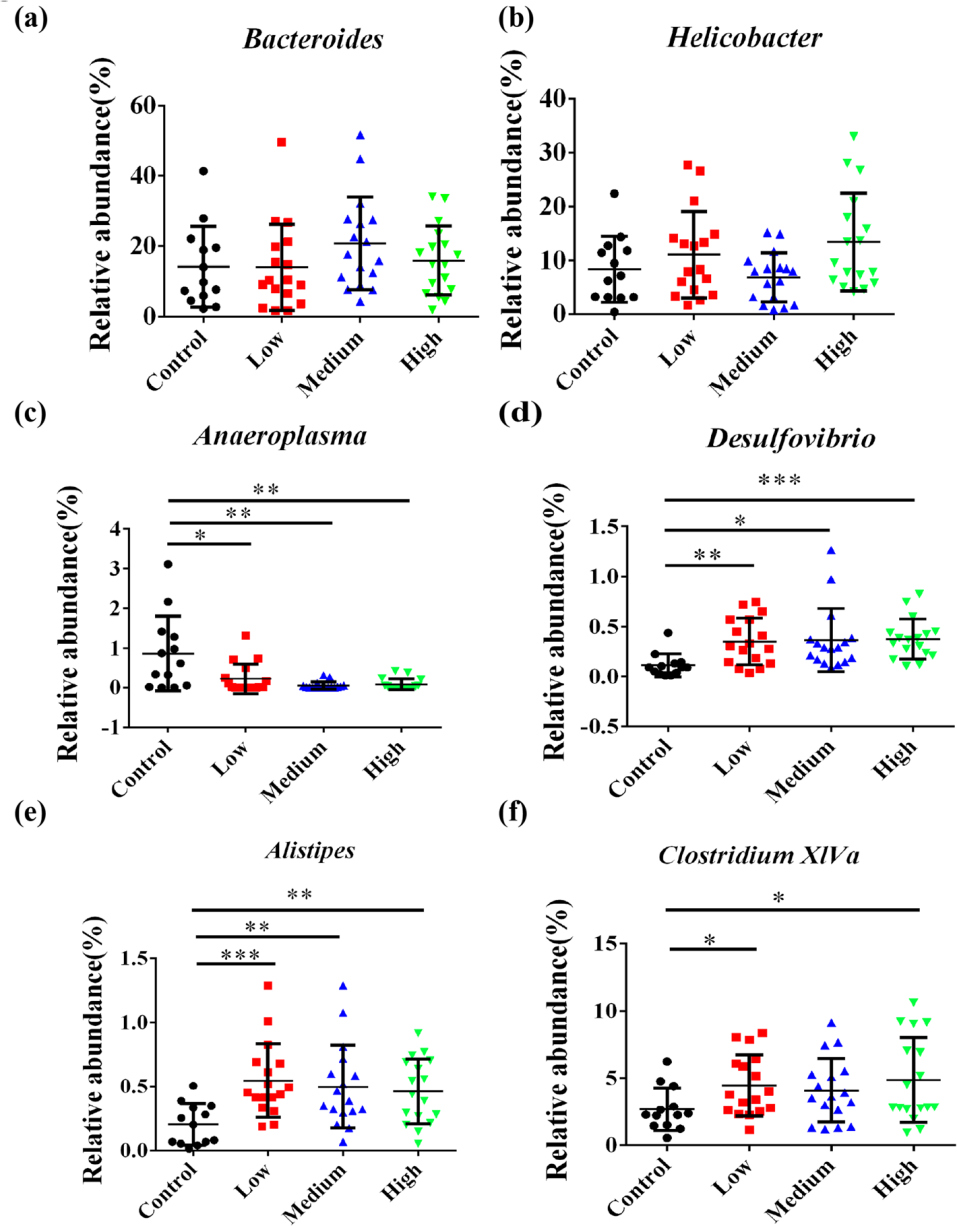
Statistical analysis of relative abundance of fecal bacteria at the genus level. **a** The abundances of *Bacteroides* were not significantly altered among four groups. **b** The abundances of *Helicobacter* showed no statistical differences among four groups. **c** The abundances of *Anaeroplasma* were significantly decreased in RbCl groups compared with the control group. **d** The abundances of *Desulfovibrio* showed statistical differences in RbCl groups compared with the control group. **e** The abundances of *Alistipes* significantly increased in RbCl groups compared with the control group. **f** The abundances of *Clostridium XlVa* were significantly higher in RbCl groups than those of the control. Data are shown as mean ± SD. *P < 0.05, **P < 0.01, ***P < 0.001

Figure 12 showed the microbial compositions with relative abundance above 5% at the genus level. The microbial community compositions were similar but relative abundances of genera varied. OTUs unclassified at the genus level were the most abundant and there were no statistical significant differences among all fecal samples. The following genera were *Bacteroides* and *Helicobacter* (the average abundances were 13.96–20.80% and 6.87–13.46%, respectively) (Table. S5). Figure 13a showed that the proportions of *Bacteroides* were no statistical differences among the four groups. *Helicobacter* showed an increasing trend in relative abundances while there were no statistical differences **(**Fig. 13b**)**. We could also get this information from heat map (Fig. S2**)**. The relative abundances of *Anaeroplasma* (Fig. 13c; *P* < 0.001) and *Desulfovibrio* (Fig. 13d; *P* < 0.001) were significantly different in various treatment groups. We observed an increase in the proportion of *Desulfovibrio* in RbCl treatment mice. The abundances of *Alistipes* (Fig. 13e; *P* < 0.01) and *Clostridium XlVa* (Fig. 13f; *P* < 0.05) were significantly higher in all the treatments than those of the control.

**Fig. 14:**
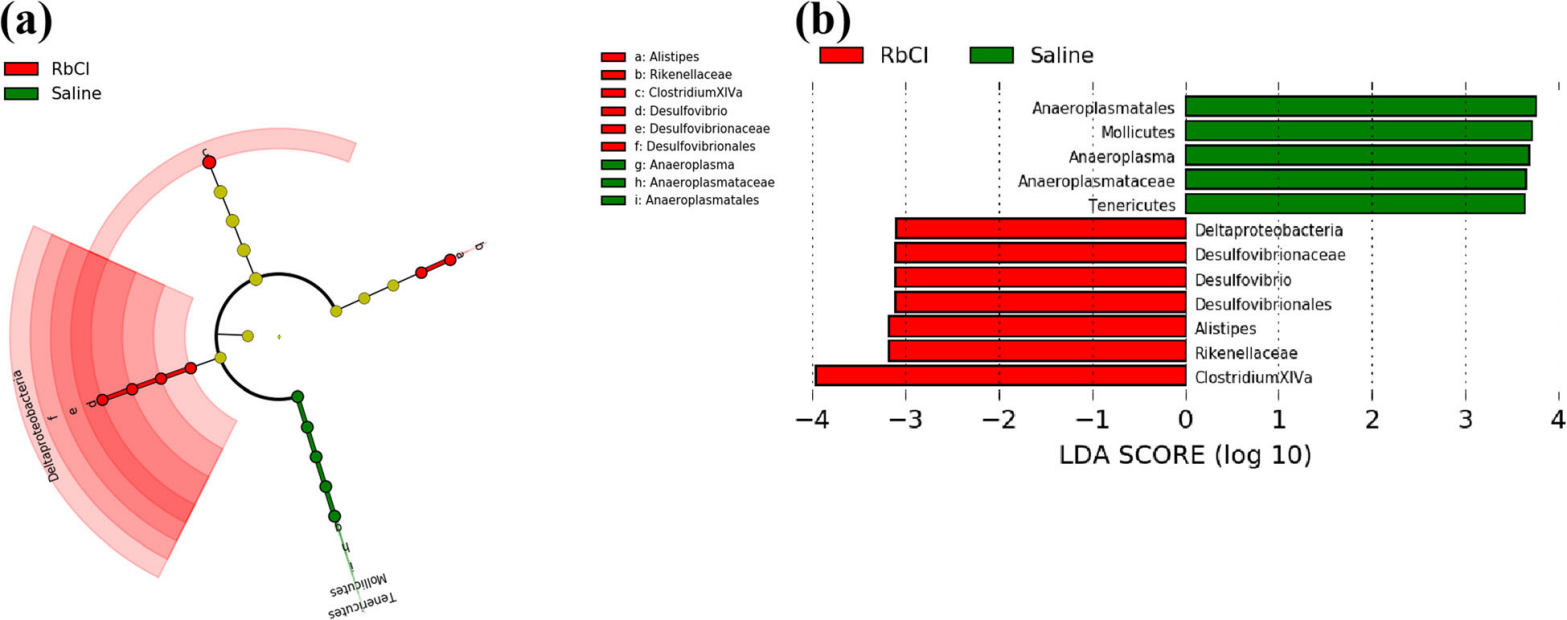
LEfSe analysis of enriched bacterial taxa in fecal microbiota between RbCl groups and the control group. **a** Taxonomic representation of statistically and biologically consistent differences between RbCl and control mice. Significant differences were represented by different colors (red and green represented the enriched microbes in the RbCl and Saline treatment groups, respectively). **b** Histogram of the LDA scores for differentially abundant genera between the two treatment groups

The LEfSe with default parameters was used to identify significant differences in relative abundances of fecal microbiota between the RbCl groups and control group. LEfSe analysis further confirmed enrichment microbes in different groups (Fig. 14a and b). The RbCl groups were significantly enriched for *Deltaproteobacteria*, *Desulfovibrionales*, *Desulfovibrionaceae*, *Desulfovibrio*, *Rikenellaceae*, *Alistipes* and *Clostridium XlVa*. The control group was enriched for *Tenericutes*, *Mollicutes*, *Anaeroplasmatales*, *Anaeroplasmataceae* and *Anaeroplasma*.

### Effect of RbCl on fecal archaea composition

**Fig. 15:**
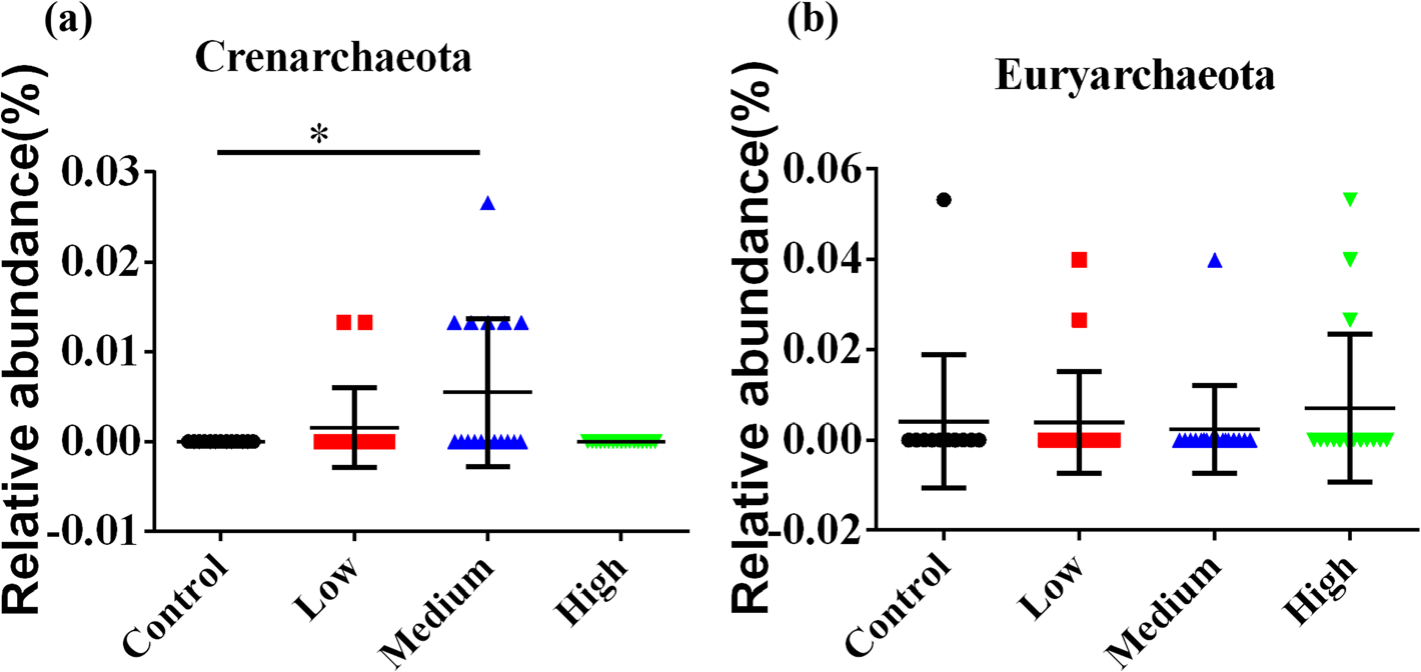
Statistical analysis of relative abundance of fecal archaea at the phylum level. **a** The abundances of *Crenarchaeota* increased in medium-dose group. **b** The abundances of *Euryarchaeota* showed no statistical differences among four groups. Data are shown as mean ± SD. *P < 0.05, **P < 0.01, ***P < 0.001

We also analyzed the abundance of various archaea in fecal samples from each treatment group. At the phylum level, the fecal archaea from 4 groups were separated into *Crenarchaeota* and *Euryarchaeota* (Table. S1). The abundance of *Crenarchaeota* was higher in middle-dose group than control group (Fig. 15a), while the abundances of *Euryarchaeota* were not significantly different among the four groups (Fig. 15b).

The fecal archaea were separated into *Thermoprotei* and *Thermoplasmata* at the class level (Table. S2). The abundance of *Thermoprotei* increased in middle-dose group compared with the control group (Fig. S3a). The abundances of *Thermoplasmata* were not significantly different among the four groups (Fig. S3b).

The relative abundances of fecal archaea in control, low-dose, middle-dose and high-dose groups at the order level were shown in Table. S3. We observed an increase in the proportion of *Sulfolobales* in middle-dose group (Fig. S4a). The relative abundances of archaea *Thermoplasmatales* were not significantly different among four groups (Fig. S4b).

At the family level, *Sulfolobaceae* (the average abundances were 0–0.01%) and *Ferroplasmaceae* (the average abundances were 0–0.01%) were observed (Table. S4**)**. The relative abundance of *Sulfolobaceae* increased in middle-dose group compared with the control group (Fig. S5a), while the abundances of *Ferroplasmaceae* demonstrated no statistical differences in control, low-dose, middle-dose and high-dose groups (Fig. S5b).

At the genus level, we observed two archaea *Sulfolobus* and *Acidiplasma* (Table. S5). Compared with the control group, the abundance of *Sulfolobus* increased in middle-dose group (Fig. S6a). The abundances of *Acidiplasma* were not significantly different among four groups (Fig. S6b).

Overall, the relative abundance of archaea in stool samples was very low. At the genus level, we only observed archaea *Sulfolobus* and *Acidiplasma*. Middle-dose RbCl could increase the relative abundance of *Sulfolobus*.

## Discussion

This study found no differences in the alpha-richness and diversity indexes, which were consistent with some reports. Getachew et al. [24] reported no significant differences in bacterial diversity and species richness between saline and antidepressant drug ketamine groups. Furthermore, study by Zhang et al. [25] had compared the gut microbiota of T2DM rats and rats treated with metformin, with no significant differences reported in alpha-richness and diversity indices. It should be noted that the diversity of bacteria was affected by several factors, including health status, age, diet, medication and so on [26]. No difference in the alpha-richness and diversity may be explained in part by the consistency of age and diet among all samples. Part of the reasons may be that RbCl did not affect the diversity of the fecal microbial communities. In addition, we found that RbCl altered the structure of fecal bacterial communities, reflected in changes in fecal microbial composition. Wei et al. [27] observed that microbiota were significantly different between healthy rats and chronic diseased rats. Moreover, Zhang et al. [28] revealed that the microbiota structure changed significantly in response to high fat diet (HFD) feeding and berberine administration. Shifts of microbiota structure were also thought to occur in Crohn’s disease patients [29]. Thus, changes in fecal microbial composition have played an important role in the progression of human diseases.

In our study, we observed that RbCl maintained the abundances of *Firmicutes, Bacteroidetes*, *Actinobacteria*, *Bacteroides* and *Helicobacter*. Chen et al. [30] reported that *Firmicutes* significantly reduced in intestinal lumen of CRC patients. In Crohn’s disease, the abundance of *Firmicutes* was also significantly decreased [31, 32]. In addition, most works showed that *Firmicutes* was the higher abundant phylum in breast tissue [33–35]. In depression patients, it was also found that the relative abundance of *Firmicutes* significantly changed [36, 37], which was related to depression through inflammation [38]. Therefore, these findings indicated that changes of *Firmicutes* were closely associated with diseases. Anticancer and anti-depressant effects of RbCl might be mediated by maintaining the abundance of *Firmicutes* in the gut. *Bacteroidetes* was non-endospore-forming anaerobes with bile resistance, accounting for more than 25% of gastrointestinal microbiota [39–41]. Proportions of *Bacteroidetes* were significantly lower in CRC rats than in healthy rats [42, 43]. Although the exact physiological implications of *Bacteroidetes* in CRC were not fully understood, it was likely that inflammatory bowel diseases were known risk factors for CRC, and a significant reduction of the phylum *Bacteroidetes* occurred in inflammatory bowel diseases [29, 44]. In addition, Jiang et al. [37] reported that *Bacteroidetes* were significantly more abundant in active-major depressive disorder subjects. The increase in *Bacteroidetes* was mainly promoted by *Alistipes.* Naseribafrouei et al. [45] reported increased abundance of *Alistipes* in the depressed subjects. Therefore, it can be inferred that changes of *Bacteroidetes* were closely associated with diseases. The *Actinobacteria*, which is comprised of gram-positive bacteria, includes 5 subclasses and 14 suborders [46]. Major depressive disorder (MDD) patients characterized by significant increase in the relative abundance of *Actinobacteria* [36]. Yang et al. [47] reported that the abundance of *Actinobacteria* was significantly higher in the depression mice. It was possible that enrichment of *Actinobacteria* was closely related to the development of depression. Exactly, RbCl did not significantly increase the abundances of *Actinobacteria*. *Bacteroides* is anaerobic, bile-resistant, non-spore-forming, gram-negative rods [48]. Changes of *Bacteroides* were assumed to be associated with metabolic diseases such as obesity and diabetes [49, 50]. In Type I diabetes mellitus patients, *Bacteroides* was significantly increased [51]. The *Bacteroides*, which was known to be associated with increased gut permeability and inflammation, was positively associated with β-cell autoimmunity. Moreover, Zhu et al. [43] reported greater genera *Bacteroides* abundance in colon cancer patients compared with controls. It was likely that *Bacteroides* produced a metalloprotease known as fragilysin, which might favor carcinogenesis. Taken together, these findings indicated that variations of *Bacteroides* were closely associated with diseases. It should be noted that RbCl did not change the proportion of *Bacteroides*. Lower abundance of *Helicobacter* was observed in gut microbiota of overall gastric cancer (GC) patients as compared to healthy controls [52]. It was possible that low proportion of *Helicobacter* contributed to the pathogenesis of GC. Exactly, RbCl did not change proportion of *Helicobacter*.

We also found RbCl significantly inhibited the abundances of *Tenericutes*, *Mollicutes*, *Anaeroplasmatales*, *Anaeroplasmataceae* and *Anaeroplasma* lineages. Yang et al. [47] reported that the abundance of *Tenericutes* significantly decreased in the depression mice. RbCl did not improve reduction of *Tenericutes*, which was consistent with reports. A previous animal study demonstrated that antidepressant drug (R)-ketamine and (S)-ketamine also did not improve the reduced proportion of *Tenericutes* [47]. Additionally, Ketamine, known to induce antidepressant effects, also significantly reduced abundances of *Tenericutes* [24]. Tully et al. [53] reported that some species of *Mollicutes* were significant pathogens in human disease. A study also found that some *Mollicutes* were associated with diseases [54]. It was worth noting that the abundances of *Mollicutes* were significantly lower after treatment with RbCl. The reduction of *Mollicutes* could decrease the pathogenesis of depression and cancers. However, one study has reported a significant reduction in the relative abundance of *Mollicutes* in MDD patients [47]. As the physiological mechanism of *Mollicutes* in depression was unclear, further studies on the relationship between depression and *Mollicutes* are needed. *Anaeroplasmatales* is an order of *Mollicutes* bacteria which do not have a cell wall [55]. Song et al. [56] found *Anaeroplasmatales* significantly increased in depression group. In addition, ketamine, known to induce antidepressant effects, significantly reduced the abundance of *Anaeroplasmatales* [24]. Exactly, the abundances of *Anaeroplasmatales* were significantly lower in RbCl groups. *Anaeroplasmataceae*, which belongs to Class *Mollicutes* and Order *Anaeroplasmatales*, is strictly anaerobic wall-less bacteria [57]. The abundance of *Anaeroplasmataceae* significantly increased in depression group [56]. Moreover, *Anaeroplasmataceae* significantly increased in patients with Crohn’s disease localized in the colon (CCD), but significantly decreased in patients with ulcerative colitis (UC) [58]. Interestingly, we observed that RbCl inhibited the proportion of *Anaeroplasmataceae*. The reduction of *Anaeroplasmataceae* could decrease the pathogenesis of depression. In the study of colon cancer, Zeng et al. [59] found that the abundance of *Anaeroplasma* increased in the HFD-azoxymethane (AOM) group. The *Anaeroplasma* is a gram-negative bacterium, which belongs to *Mollicutes* class, *Tenericutes* phylum. *Anaeroplasma* was opportunistic pathogens which elicited various host immune responses in numerous human diseases including colon cancer [60, 61]. Interestingly, the results of RbCl inhibited the proportion of the bacteria.

Expressions of sulfate-reducing bacteria (SRB) including *Deltaproteobacteria*, *Desulfovibrionales*, *Desulfovibrionaceae* and *Desulfovibrio* were significantly higher in RbCl groups than control group. *Deltaproteobacteria* belonging to *Proteobacteria* is sulfate-reducing bacteria [62]. Hydrogen sulfide (H_2_S) produced by SRB was a product of sulfate reduction [63]. H_2_S could lead to chronic inflammation and imbalance between cellular proliferation, apoptosis and differentiation by damaging the intestinal epithelium [64]. Reports showed that *Deltaproteobacteria* was possibly associated with CRC [65, 66]. Jin et al. [67] reported that *Deltaproteobacteria* was commonly pathogenic bacteria in the intestine. *Desulfovibrionales*, belonging to *Deltaproteobacteria*, is also a sulfate-reducing bacteria that can reduce sulfur to produce hydrogen sulfide (H_2_S) [62]. *Desulfovibrionaceae*, which was the main biological source of hydrogen sulfate (H_2_S), involved in a wide range of physiological processes by influencing cellular signaling pathways and sulfhydration of target proteins [68, 69]. Zhang et al. [70] reported that the proportion of *Desulfovibrionaceae* increased in animal models of metabolic syndrome. *Desulfovibrio* could also produce H_2_S by reducing sulfate [71]. H_2_S derived from *Desulfovibrio* was associated with gastrointestinal disorders, such as UC, Crohn’s disease, and irritable bowel syndrome [68]. Besides, Hale et al. [72] also reported that *Desulfovibrio* produced metabolites such as secondary bile acids, which may catalyze the formation of colorectal cancer. However, it should be noted that the proportions of sulfate-reducing bacteria were promoted by RbCl. RbCl led to the enrichment of sulfate-reducing bacteria which could cause inflammation directly or indirectly in mice. It was likely that RbCl used as antigen in healthy mice which could elicit immune responses.

In addition, RbCl significantly increased the abundances of *Rikenellaceae*, *Alistipes* and *Clostridium XlVa*. Wu et al. [73] found that the abundance *Rikenellaceae* decreased in the colitis-associated colorectal cancer (CAC) group compared with control group. Alkadhi et al. [74] also reported that the proportion of *Rikenellaceae* reduced in CAC mice. In addition, the report found that *Rikenellaceae* was overrepresented in healthy control subjects [36]. Following RbCl treatment, the abundance of *Rikenellaceae* increased in the present study. Therefore, the increase in *Rikenellaceae* abundance could accelerate the antitumor efficacy of RbCl. *Alistipes*, which belongs to *Bacteroidetes*, is present in the human intestinal tract [75]. *Alistipes* was indole-positive and may thus influence tryptophan availability [76]. In our results, RbCl promoted the abundance of *Alistipes*. As tryptophan was also the precursor of serotonin, enrichment of *Alistipes* might affect serotonergic system by interfering with tryptophan metabolism. *Clostridium XlVa*, belonging to *Firmicutes* phylum, produces short-chain fatty acids (SCFAs) [77]. The SCFAs produced in the gut are mainly acetate, butyrate and propionate [78]. SCFAs could modulate cell functions either by inhibiting histone deacetylase activity, or through the activation of ‘metabolite-sensing’ G-protein coupled receptors (GPCRs) such as GPR43 and protect the integrity of epithelial barrier [79–81]. RbCl promoted the abundance of *Clostridium XlVa*. The increase in abundance *Clostridium XlVa* could alleviate the pathogenesis of depression and cancers. *Clostridium XlVa* was significantly lower in CRC patients than healthy subjects [77]. *Clostridium XIVa* was overrepresented in healthy control subjects [36, 82].

Regarding the composition of archaea, the abundances of *Crenarchaeota*, *Thermoprotei*, *Sulfolobales*, *Sulfolobaceae* and *Sulfolobus* lineages increased in middle-dose RbCl groups. *Crenarchaeota* was originally considered to grow in habitats characterized by high temperature, high salinity, or an extreme pH. Later studies found that *Crenarchaeota* also seem to occur ubiquitously in temperate or cold aquatic [83] and terrestrial environments [84]. The presence of *Crenarchaeota* in intestinal tracts was reported by Friedrich et al. [85]. In addition, Rieu-Lesme et al. [86] suggested that *Crenarchaeota* was present in the microbiota of the human digestive ecosystem. *Thermoprotei*, the crenarchaeal class, consists solely of obligate thermophiles. Thermophiles were well-known for participating in rampant lateral gene transfer (LGT) [87, 88]. It was likely that the nature of their extreme environments encouraged the exchange of genetic material. *Thermoprotei* mostly occurred in the marine environment [89]. However, report showed that *Thermoprotei* was observed to have an appreciably higher representation in healthy child [90]. Interestingly, the proportion of *Thermoprotei* was promoted by middle-dose RbCl in this study. *Sulfolobales*, a monophyletic group within the *Crenarchaeota*, is thermophilic sulfur-metabolizing archaea [91]. The report found that *Sulfolobales* was present in human feces sample [86]. The family *Sulfolobaceae* is composed of extreme thermoacidophiles that are found in terrestrial environments [92]. The *Sulfolobaceae* could produce bacteriocin, which played an important role in microbial interaction or microbe-environment interactions, and therefore improved their adaptation in extreme environments [93]. Enrichment of *Sulfolobaceae* promoted by middle-dose RbCl may be beneficial in combating disease-related adverse environments. The genera *Sulfolobus*, which belongs to *Sulfologaceae*, grows at low pH (2–3) and high temperature (70–85 °C) [94, 95]. The acidophilic and thermophilic properties of *Sulfolobus* offered many obvious advantages for industrial applications [96, 97]. In addition, *Sulfolobus* was able to reduce ferric iron when growing on elemental sulfur as an energy source [98].

Furthermore, RbCl maintained the abundances of archaea *Euryarchaeota*, *Thermoplasmata*, *Thermoplasmatales*, *Ferroplasmaceae*, *Acidiplasma* lineages. *Euryarchaeota*, one of the four major divisions of archaea, contributed substantially to global energy cycling [99]. *Euryarchaeota* was detected in marine picoplankton [100, 101] and in coastal salt marsh and continental shelf sediments [102]. *Methanobrevibacter smithii*, which belonged to *Euryarchaeota* phylum, was a major archaeal player in human gut system [103]. A few studies confirmed that *M. smithii* was probably involved in inflammatory bowel disease (or Crohn’s disease), irritable bowel syndrome, colorectal cancer, and obesity [104, 105]. *Methanobrevibacter oralis*, belonging to *Euryarchaeota* phylum, was the predominating methanogenic species in the oral cavity [103]. *M. oralis* was identified in apical periodontitis [106]. Therefore, these findings proved that *Euryarchaeota* might play key roles for human health and disease. However, the proportions of *Euryarchaeota* did not significantly change after RbCl treatment. *Thermoplasmata* was affiliated with *Euryarchaeota* phylum. Auguet et al. [107] showed that *Thermoplasmata* represented important component of soil microbial communities. In the human body, Li et al. [108] found that *Thermoplasmata* was not the predominant archaeons in the subgingival dental plaque and *Thermoplasmata* was closely correlated with chronic periodontitis. Following RbCl treatment, the abundance of *Thermoplasmata* did not significantly change. Horz et al. [109] found that *Thermoplasmatales* existed in the human oral cavity. He et al. [110] reported that *Thermoplasmatales* was also observed in healthy subjects, but the abundance of *Thermoplasmatales* increased in individuals with periodontitis. It was possible that enrichment of *Thermoplasmatales* contributed to the pathogenesis of periodontitis. Exactly, RbCl did not improve the enrichment of *Thermoplasmatales*. The *Ferroplasmaceae* is represented by cell wall-deficient, acidophilic, facultatively anaerobic and iron-oxidizing archaea [111]**.** As iron oxidizers, the family *Ferroplasmaceae* may contribute to the cycle of iron and sulfur [112]. It was likely that *Ferroplasmaceae* was involved in the pathogenesis of diseases through oxidizing iron. Thus, further studies on the relationships between diseases and *Ferroplasmaceae* are needed. Interestingly, RbCl did not significantly change the abundance of *Ferroplasmaceae. Acidiplasma*, which belongs to the family *Ferroplasmaceae*, order *Thermoplasmatales*, phylum *Euryarchaeota*, is a novel acidophilic, cell-wall-less archaeon [113]. The genera *Acidiplasma* included two species, namely *Acidiplasma aeolicum* and *Acidiplasma cupricumulans* [112]. *Acidiplasma aeolicum* and *Acidiplasma cupricumulans* were isolated from the hydrothermal pool located on Vulcano Island (Italy) and chalcocite/copper-containing heaps (Myanmar), respectively [113]. It should also be noted that there were no reports on the relationships between *Acidiplasma* and diseases. In our results, *Acidiplasma* was observed in stool samples and its abundances were not significantly changed by RbCl.

Some reports found Rb could be used as anticancer and anti-depressant drugs. The mechanisms of Rb against cancer and neurological disease remain unclear. Microbiota may participate in the pathogenesis of depression through the brain-gut-microbiota axis [114]. Serotonin (5-HT) is a critical signaling molecule in the brain-gut-microbiota axis [115]. The accumulation of 5-HT and the rate of synthesis of 5-HT in the brain were enhanced by intraperitoneal administration of RbCl [116]. In the present study, *Clostridium XlVa*, SCFAs producing bacteria, was significantly promoted by RbCl. SCFAs could promote colonic 5-HT production [117, 118]. Enrichment of *Alistipes* promoted by RbCl might disrupt the intestinal serotonergic system by affecting tryptophan metabolism. Therefore, the microbes might partly promote the anticancer and anti-depressant effects of RbCl via brain-gut-microbiota axis.

## Conclusions

In summary, our results revealed RbCl significantly altered fecal microbial composition. RbCl maintained the abundances of dominant bacteria. However, RbCl significantly altered the abundances of less richness microbes. Changes in fecal microbes might in part contribute to the anticancer or anti-depressant effects of RbCl. Clearly, further functional analysis of the role of specific fecal microorganisms and their interactions with brain-gut-microbiota axis is expected.

## Methods

### Experimental animals and experimental design

Three-week old male Swiss mice used as experimental animals (license number SCXK (Xiang) 2016–0002) were purchased and raised in the Laboratory Animal Science Department (LASD) of Central South University with Specific Pathogen Free (SPF) level environment. The living environment of the mice was of constant temperature (20 ± 2 °C), constant humidity (50 ± 10%), and free access to water and food. Mice were strictly controlled in normal biological rhythms and the light and dark environments were 12 h, respectively. All animal experiments in this study were approved by the Animal Breeding and Committee of the Department of Laboratory Animal Science of Central South University and were strictly evaluated in accordance with the Regulations on Animal Management of Central South University. The mice were kept in the LASD for a week without any treatment to adapt to the environment. Sixty-four mice were randomly assigned into four groups: one was blank control group which was intervened with normal saline (*n* = 13), and the other three groups were divided into low-dose (*n* = 17), middle-dose (*n* = 17), and high-dose group (*n* = 17) according to the different RbCl dosage (20 mg/L (0.17 mmol/L), 50 mg/L (0.41 mmol/L), 100 mg/L (0.83 mmol/L), respectively). Five or four mice were randomly placed in each mouse cage. The mice of the above experimental groups were intragastrically administered of RbCl in 0.2 mL twice per day for 6 consecutive weeks. During this period, the mice were weighed weekly.

### Fecal samples collection and properties analysis

After 6 weeks of drug treatments, the mice to be sampled were placed on a clean ultra-clean bench with sterile filter papers for taking stool samples. The fecal samples were collected into the sterile tubes immediately after defecation. The tubes were marked and snap frozen in liquid nitrogen. All mice were sacrificed by pentobarbital overdose (60 mg/kg) in the ultra-clean workbench. The kidneys, heart, lungs, pancreatic, spleen, stomach and liver were rapidly excised from mouse and weighed.

### Fecal DNA extraction and sequencing

Each fecal sample (approximately 0.2 g) was used for total gut microbiome DNA extraction with QIAGEN QIAamp kit. Extractions were performed according to specific operating instructions. The extracted total genomic DNA was detected by agarose gel electrophoresis and qualified DNA samples were used in subsequent experiments. PCR amplification and library preparation were performed using 515 F (5′-GTGCCAGCMGCCGCGGTAA-3′) and 806R (5′-GGACTACHVGGGTWTCTAAT-3′) primers to target the V4 region of the 16S rRNA gene. The PCR products for each sample were subjected to electrophoresis at a voltage of 100 V for about 1 h using a 2% agarose gel. The target band was recovered by tapping under UV light, and E.Z.N.A.TM Gel Purification Kit (OMEGA Bio-Tek Inc., USA) was used for product purification. The purified product was quantified using a Nanodrop spectrophotometer (ND-1000 spectrophotometer, Wilmington, USA). Illumina MiSeq (Illumina, San Diego, CA) sequencing required the library constructed from the mixture of 200 ng of each purified product.

### Data processing and sequence analysis

The MiSeq sequencing data were analyzed using the Galaxy pipeline developed by Prof. Zhou’s lab (http://zhoulab5.rccc.ou.edu/) at University of Oklahoma. The resulting sequences were further filtered based on quality score and sequence length. To merge the paired-end reads into full-length amplicon sequence, the FLASH software tool was used based on overlapping bases. The sequences were clustered into operational taxonomic units (OTU) at or above 97% identity. According to previous reports, OTUs reaching 97% similarity were used to analyze alpha diversity (Shannon and Simpson), and richness (Ace, Chao and Sobs) [119, 120].

### Statistical analysis

IBM SPSS Statistics 19.0 software was used for statistical analysis. Since comparison was performed between two groups (saline and low, middle, high, respectively), Student T-test was applied for detecting significant differences in specific measured parameters. All values were expressed as the mean ± standard deviation (SD). Probability values of less than 0.05 were considered to show a statistical significance. Microbiota community diversity and richness were analyzed using vegan package and R software (version 3.5.1). LEFSe (Linear discriminant analysis effect size), CPCoA (constrained principal coordinate analysis) and Heatmap plot were performed on ehbio BioPharm platform (http://www.ehbio.com).

## Supplementary Information

**Additional file 1.** Venn analysis of shared and unique OTUs.

**Additional file 2.** Heat map of fecal microbe at the genus level.

**Additional file 3.** Statistical analysis of relative abundance of fecal archaea (class).

**Additional file 4.** Statistical analysis of relative abundance of fecal archaea (order).

**Additional file 5.** Statistical analysis of relative abundance of fecal archaea (family).

**Additional file 6.** Statistical analysis of relative abundance of fecal archaea (genus).

**Additional file 7.** Relative abundances of all microorganisms at the phylum level.

**Additional file 8.** Relative abundances of all microorganisms at the class level.

**Additional file 9.** Relative abundances of all microorganisms at the order level.

**Additional file 10.** Relative abundances of all microorganisms at the family level.

**Additional file 11.** Relative abundances of all microorganisms at the genus level.

## Data Availability

All sequence data were deposited into the NCBI Sequence Read Archive database (accession No. PRJNA630020). All the 16S rDNA sequences of 64 samples have been upload to NCBI database (SRA accession No. SRR11671062 - SRR11671125).

## Abbreviations

Rb: rubidium
RbCl: Rubidium chloride
CPCoA: Constrained principal coordinate analysis
HFD: High fat diet
CRC: Colorectal cancer
MDD: Major depressive disorder
GC: Gastric cancer
CCD: Crohn’s disease localized in the colon
UC: Ulcerative colitis
AOM: Azoxymethane
SRB: Sulfate-reducing bacteria
H_2_S: Hydrogen sulfide
CAC: Colitis-associated colorectal cancer
SCFAs: Short-chain fatty acids
GPCRs: G-protein coupled receptors
LGT: Lateral gene transfer
5-HT: Serotonin
LASD: Laboratory Animal Science Department
SPF: Specific Pathogen Free
OUT: Operational taxonomic units
LEFSe: Linear discriminant analysis effect size
ANOVA: One-way analyses of variance
