# Supplementary figures and images for: Rubidium chloride modulated the fecal microbiota community in mice
Source: BMC Microbiol. 2021 Feb 15;21:46. doi: 10.1186/s12866-021-02095-4 (PMC7885239; doi:10.1186/s12866-021-02095-4)

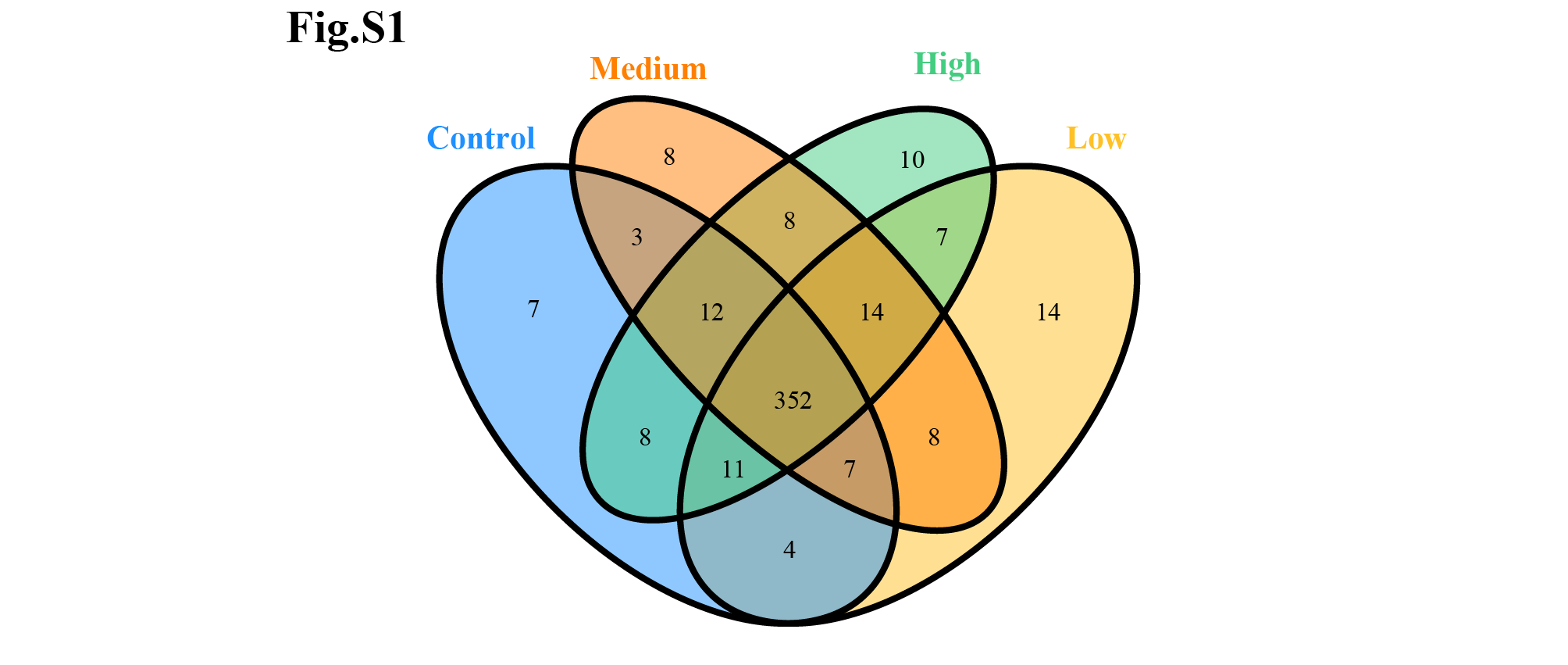

Supplement: Supplementary file 1 — Additional file 1. Venn analysis of shared and unique OTUs. [file 12866_2021_2095_MOESM1_ESM.tif]

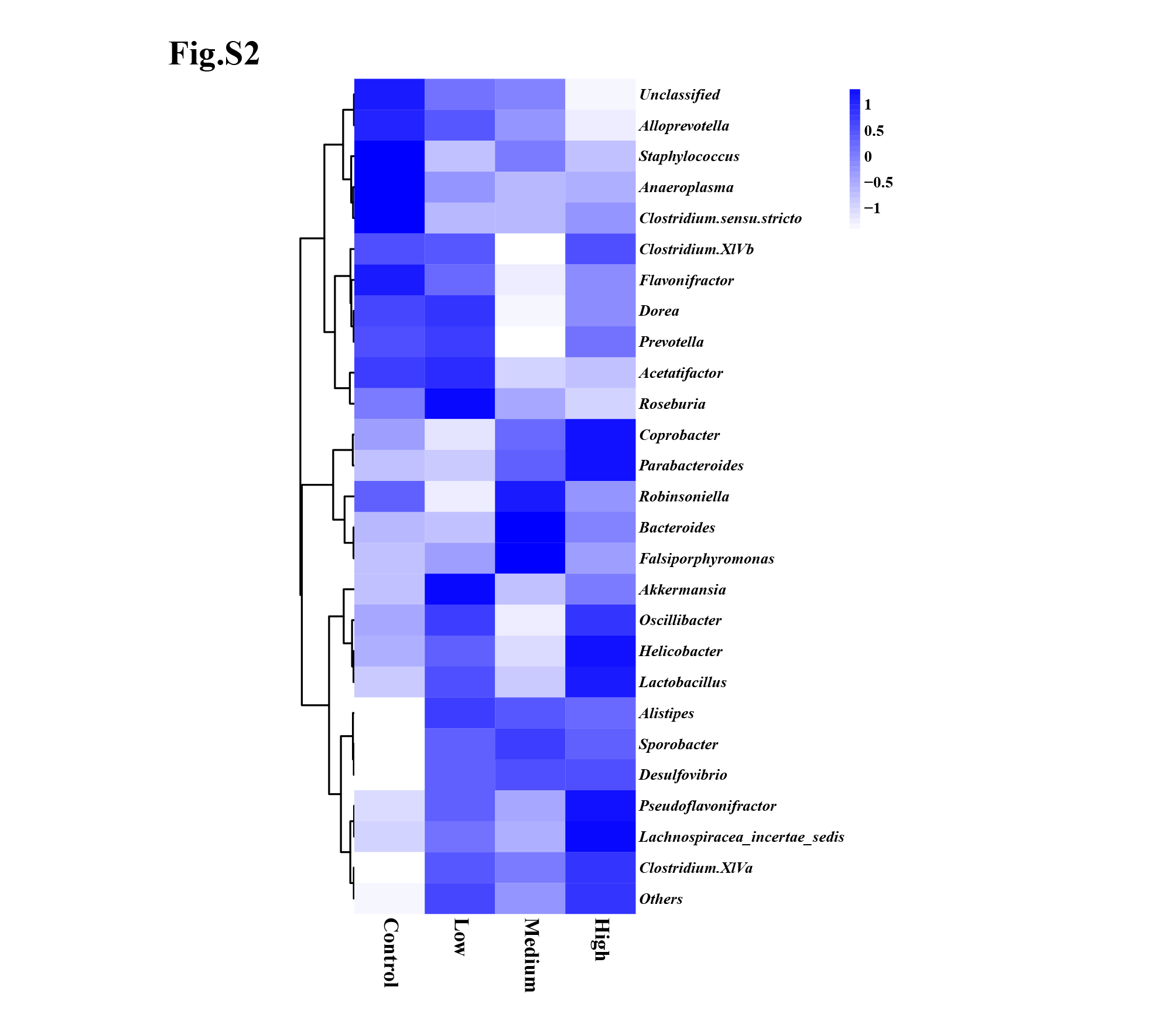

Supplement: Supplementary file 2 — Additional file 2. Heat map of fecal microbe at the genus level. [file 12866_2021_2095_MOESM2_ESM.tif]

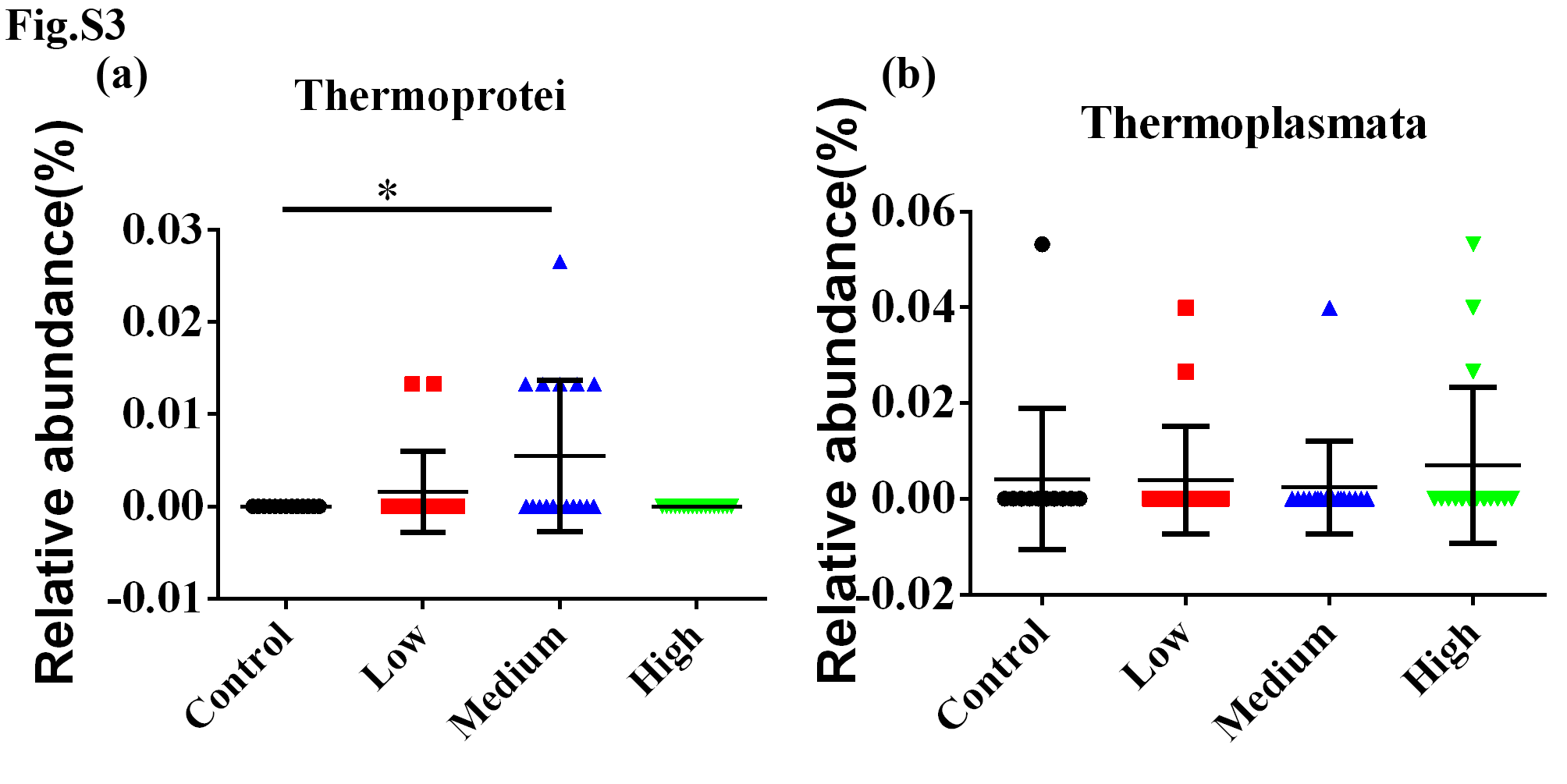

Supplement: Supplementary file 3 — Additional file 3. Statistical analysis of relative abundance of fecal archaea (class). [file 12866_2021_2095_MOESM3_ESM.tif]

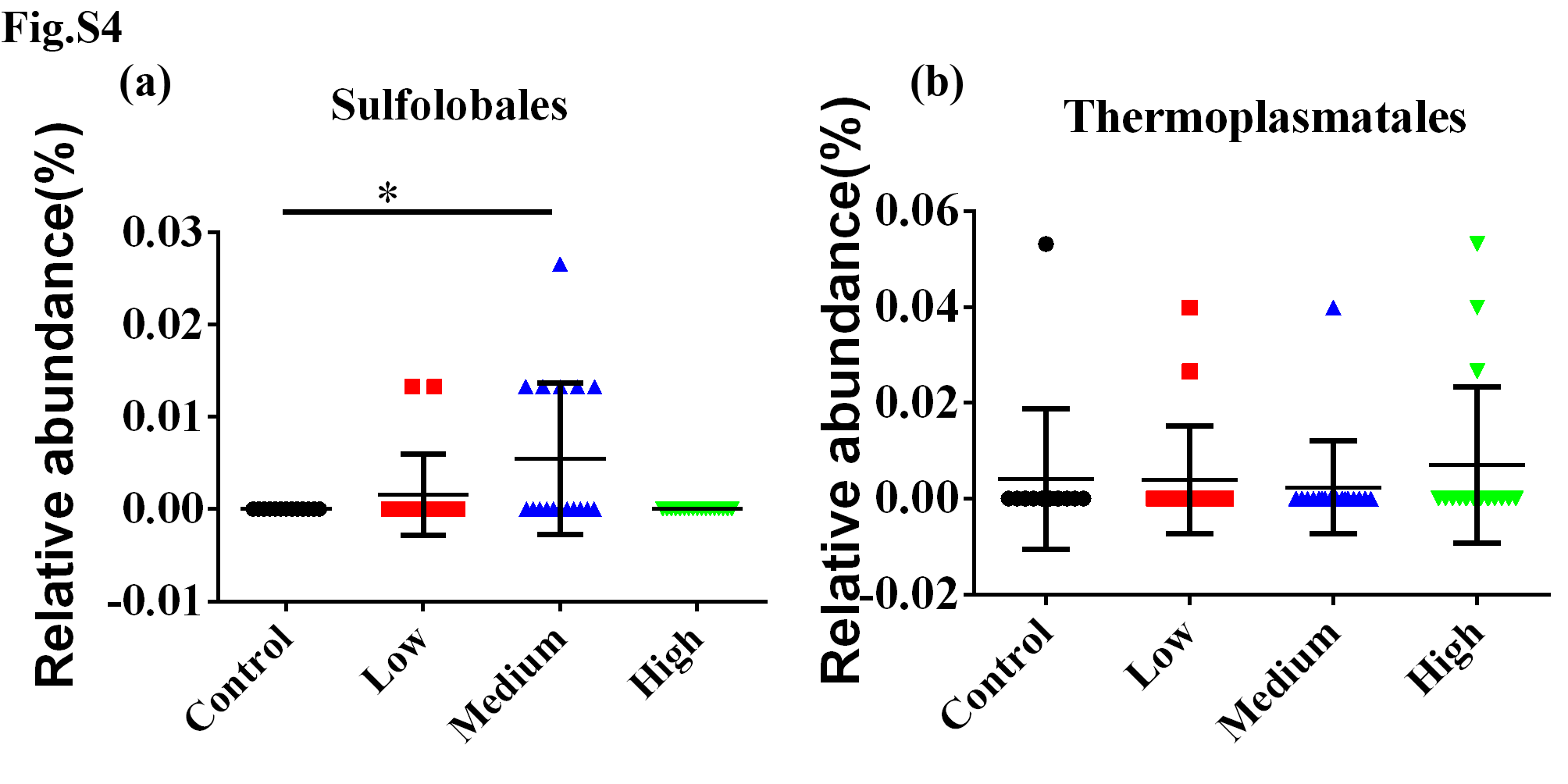

Supplement: Supplementary file 4 — Additional file 4. Statistical analysis of relative abundance of fecal archaea (order). [file 12866_2021_2095_MOESM4_ESM.tif]

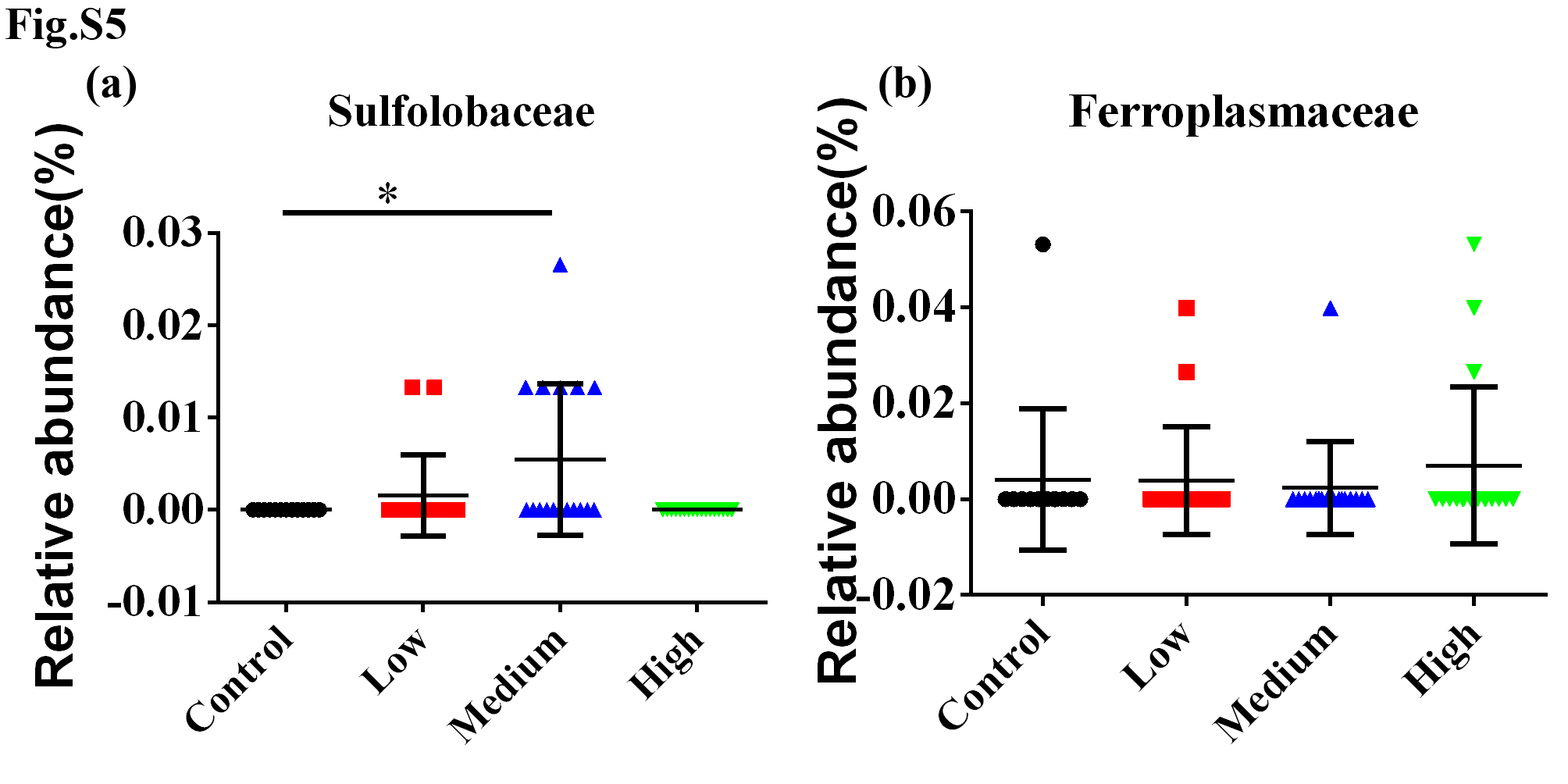

Supplement: Supplementary file 5 — Additional file 5. Statistical analysis of relative abundance of fecal archaea (family). [file 12866_2021_2095_MOESM5_ESM.tif]

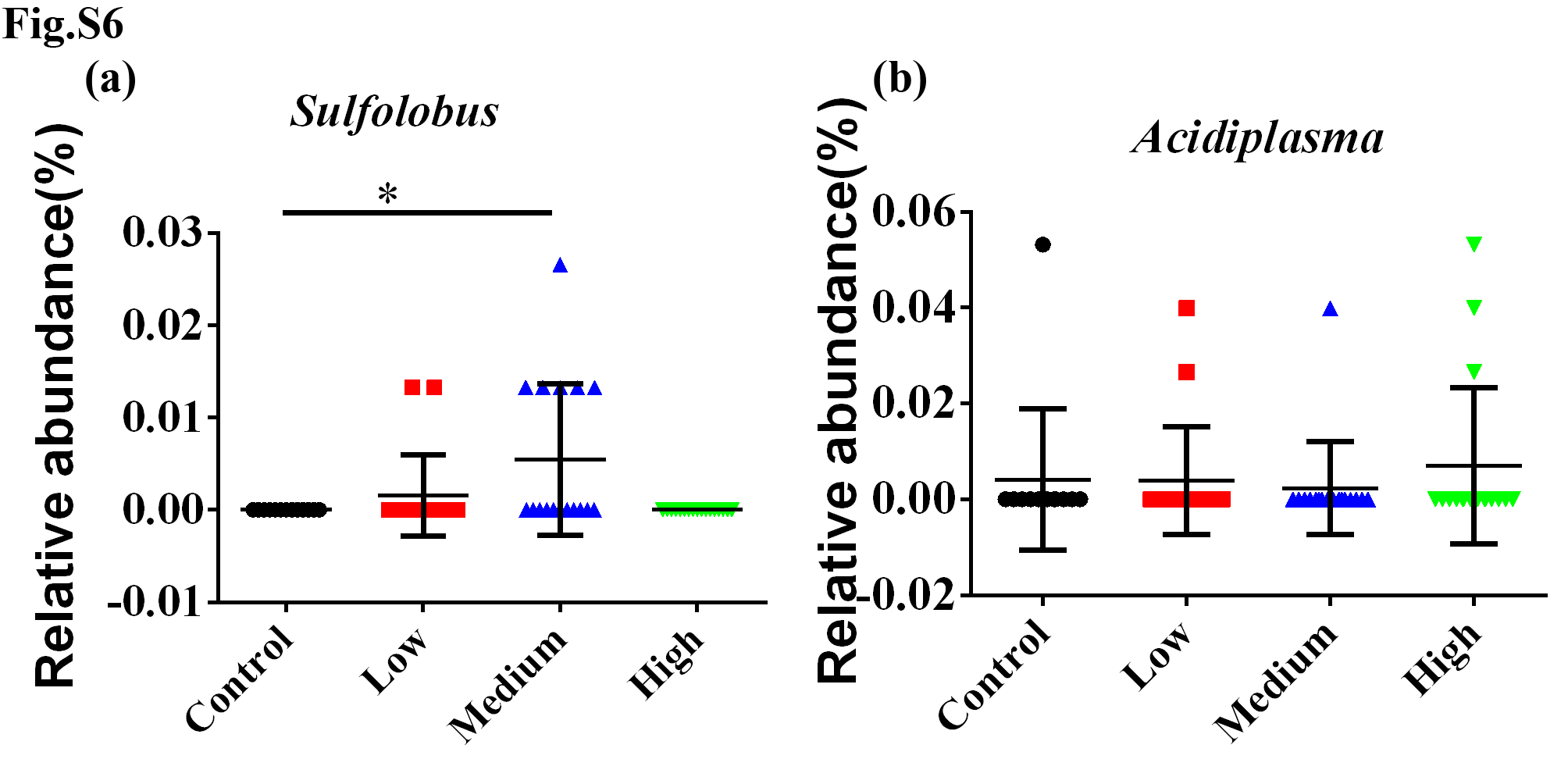

Supplement: Supplementary file 6 — Additional file 6. Statistical analysis of relative abundance of fecal archaea (genus). [file 12866_2021_2095_MOESM6_ESM.tif]
